# ‘Some Gave It to Cow, Others to Woman’ – Traditional Knowledge and Use of Tuberous Orchids in Hungarian Communities in the Eastern Carpathians (Romania)

**DOI:** 10.3390/plants15152322

**Published:** 2026-07-28

**Authors:** Attila Molnár V., Tünde Hunyadi, Jenő Nagy, Diána Gszellmann

**Affiliations:** HUN-REN–UD Conservation Biology Research Group, Department of Botany, University of Debrecen, Egyetem sq. 1, H-4032 Debrecen, Hungaryjenonagy.off@gmail.com (J.N.);

**Keywords:** aphrodisiacs, Doctrine of Signatures, ethnobotany, ethnoveterinary, folk taxonomy, knowledge erosion, Orchidaceae, Transylvania, Vernacular plant names

## Abstract

The tubers of terrestrial orchids have been used for nutritional, medicinal, and aphrodisiac purposes across Europe and Western Asia for centuries. Historical sources from Hungary and Transylvania indicate that tuberous orchids, collectively known as *bergőburján* in the Gyimes region of the Eastern Carpathians, were believed to possess aphrodisiac properties and were used in both human and veterinary medicine. This exploratory study documents contemporary knowledge, nomenclature, beliefs, and uses associated with tuberous orchids among Hungarian-speaking communities in the Eastern Carpathians of Romania. A total of 104 semi-structured interviews were conducted in seven settlements of the Csík and Gyimes regions in 2023. Knowledge of *bergőburján* was much more frequently recorded in Gyimes (54/85 respondents; 63.5%) than in the sampled Csík settlements (1/19 respondents; 5.3%). Statistical analyses revealed that informants from Gyimes recognised and named significantly more orchid species, while older respondents demonstrated substantially greater orchid-related knowledge than younger individuals. Farmers also recognised more orchid species than manual workers. More than one-third of respondents familiar with *bergőburján* associated it with aphrodisiac effects, and over half reported its former use as a stimulant for humans and livestock. Although the use of *bergőburján* largely ceased decades ago, a distinctive body of traditional knowledge persists. Our results indicate that *bergőburján*-related knowledge has persisted among older community members despite the disappearance of its practical use. However, generational differences suggest that the intergenerational transmission of this traditional ecological knowledge is weakening.

## 1. Introduction

**Figure 1:**
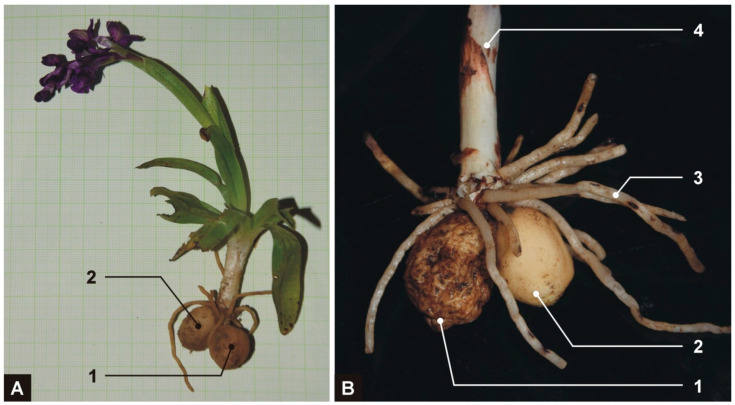
The paired, rounded tubers of some terrestrial orchid genera (e.g., *Anacamptis*) resemble testicles. (**A**) Whole plant. (**B**) Underground part. 1 – previous year’s tuber (in Hungarian: *‘anyagumó’,* i.e., mother-tuber); 2 – current year’s tuber (‘leánygumó’, i.e., daughter-tuber); 3 – root; 4 – stem. Credit: A.M.V.

The use of terrestrial orchids by humans has a long and well-documented history extending from Antiquity to the present day. Particular attention has been paid to species possessing paired underground tubers (Figure 1), whose resemblance to human testes inspired medicinal and symbolic interpretations under the Doctrine of Signatures. According to this concept, plants resembling specific body parts were believed to possess therapeutic effects on the corresponding organs or functions [1,2,3]. The ancient Greek term Oρχις (*Orchis*), meaning ’testicle’, gave rise to both the generic name *Orchis* and the name of the entire orchid family (Orchidaceae). Since classical times, orchid tubers have frequently been associated with fertility, virility and aphrodisiac properties [2,3,4].

Traditional ecological knowledge (hereafter: TEK) is not simply a collection of utilitarian observations, but a culturally transmitted and socially embedded knowledge system shaped by everyday practice, oral communication, memory and local classification. Symbolic plant uses may persist across generations when they are linked to visually salient morphological traits, memorable vernacular names, practical applications and recurring narratives within a community. In such cases, plant morphology, language and social memory may reinforce one another, allowing particular interpretations of plants to survive even after their practical use has declined. Tuberous orchids provide a suitable example of this process, because their paired underground organs, vernacular names and historical associations with fertility and sexual potency form a coherent symbolic complex that can be transmitted through both practical knowledge and oral tradition.

The collection and use of orchid tubers have been documented throughout large parts of Europe and Western Asia. Tuberous orchids have traditionally been harvested for medicinal purposes and as food, particularly in the Balkans, Anatolia, the Caucasus and the Middle East [5,6,7,8,9,10,11,12,13,14,15,16,17]. Recent reviews have highlighted both the cultural significance of orchid use and the conservation concerns associated with harvesting wild populations [18,19,20,21]. Most previous ethnobotanical studies on tuberous orchids have focused on regions where orchid tubers are still collected, consumed or traded, particularly in relation to *salep* production, traditional medicine and conservation conflicts arising from harvest pressure. By contrast, less attention has been paid to communities where former orchid use has largely disappeared from everyday practice, but may persist in vernacular nomenclature, social memory, symbolic associations and ethnoveterinary knowledge. Although previous ethnobiological studies have documented rich plant knowledge and traditional land-use practices among Hungarian-speaking communities in the Eastern Carpathians, quantitative, interview-based documentation of orchid-specific TEK in the Gyimes communities has remained limited. The present study therefore contributes to orchid ethnobotany by focusing specifically on tuberous orchids and the historically documented folk taxon *bergőburján*, linking historical records with contemporary interview data, and assessing whether associated names, beliefs and reported former uses persist in present-day local knowledge.

Historical Hungarian sources indicate that tuberous orchids were known and occasionally utilised in parts of the Hungarian-speaking area, although the geographical scope and ethnographic specificity of these records vary considerably. Forgó [22] reported in 1817 the consumption of orchid tubers during periods of famine, while Fäller [23] and Augustin et al. [24] documented their medicinal applications. Several authors also recorded beliefs concerning the aphrodisiac properties of orchid tubers. Veszelszki [25] described the contrasting effects attributed to fresh and withered tubers in 1798. Diószegi [26] characterised orchids as “arousing love” in 1813 and Berde [27] mentioned their use against impotence. Particularly noteworthy is the work of Wagner [28] from 1899, who recorded the vernacular name *bergőburján* in the Eastern Carpathians and noted that orchid tubers were used for both humans and livestock because they were believed to stimulate sexual activity. This record is geographically the most relevant historical source for the present study, because it refers to the former Csík County, including the Gyimes region where our contemporary interviews were conducted. By contrast, several other early sources cited above reflect the broader Hungarian botanical, linguistic or ethnomedical literature and should not be regarded as evidence for a uniform distribution of orchid-related beliefs across the whole Hungarian-speaking area.

Beliefs concerning the aphrodisiac properties of orchid tubers recur throughout Hungarian historical sources. These records, spanning more than 150 years, provide an important historical and linguistic context for interpreting contemporary local knowledge. They do not demonstrate a continuous and geographically uniform tradition across the entire Hungarian-speaking area, but they offer a relevant comparative framework for assessing whether orchid-related names, beliefs and uses documented in earlier sources are still reflected in present-day knowledge in Gyimes. To the best of our knowledge, all relevant Hungarian historical sources that explicitly mention tuberous orchids in relation to aphrodisiac interpretations are summarised in Table 1. These sources differ in their geographical specificity, but together they provide the historical and linguistic background for interpreting the contemporary *bergőburján* data.

The name *bergőburján* was first published by Wagner [28] from the former Csík County, including the Gyimes region of the Eastern Carpathians, in 1899. The verbs ‘bereg’, ‘bërëg’, ‘berreg’, ‘bërrëg’, ‘börrög’ denote ‘to mate’ in Transylvanian Hungarian [29], while the suffix ‘burján’ is still used in Transylvania to mean ‘weed’ or ‘wild herbaceous plant’. Among Csángó people (Hungarian ethnic group) living in the Gyimes region, species from several orchid genera (*Dactylorhiza*, *Gymnadenia*, *Nigritella*, and *Platanthera*) are still referred to as *bergőburján* [30] (p. 125), while those from the settlement of Háromkút consistently refer to *Nigritella rubra* K. Richt. [31] (p. 45), which is also known as ‘*vitézvér*’ (i.e., ‘valiant blood’). Additionally, a perceived aphrodisiac effect may underlie the old names ‘nőszőfű’ (mating-herb) and ‘embererőfű’ (herb of man-power), as well as the name ‘kos-mony’ (testicle of rams), as summarised by Rácz [29,32].

The Gyimes region of the Eastern Carpathians in Romania is an area where previous ethnobiological studies have documented the persistence of extensive local ecological knowledge and traditional land-use practices. The landscape is characterised by species-rich semi-natural grasslands and hay meadows that harbour numerous orchid species [31,33,34,35]. Such meadows can be regarded as biocultural landscapes, because their high biodiversity is closely linked to long-term, low-intensity management, including mowing, grazing and local hay-making practices. In these systems, ecological knowledge is not separated from everyday work, but is embedded in the recognition of plants, habitat types, seasonal changes and culturally meaningful species. Vernacular plant names and narratives may therefore reflect not only botanical recognition, but also the social and symbolic importance of particular plants within the local landscape.

Previous ethnobiological studies have demonstrated that local Hungarian-speaking communities retain extensive knowledge of plants, habitats and traditional land-use practices [30,33,34,35]. However, little is known about the contemporary status of orchid-related knowledge and whether historical traditions associated with *bergőburján* have survived into the twenty-first century.

TEK is increasingly recognised as vulnerable to rapid erosion due to socio-economic changes, urbanisation and shifts in land use. Documenting such knowledge is therefore important not only from cultural and historical perspectives but also for understanding how local communities perceive and interact with biodiversity. Orchids are particularly suitable subjects for such investigations because of their conspicuous appearance, cultural significance and strong associations with traditional beliefs. Similar historical ethnobotanical approaches have recently shown that Hungarian archival sources can preserve substantial information on former plant distributions, vernacular nomenclature, ethnomedicinal and ethnoveterinary practices, and local ecological knowledge, as demonstrated for the highly toxic wetland species *Cicuta virosa* in Hungary [36].

The aims of this study were therefore to investigate the contemporary knowledge of orchids among Hungarian-speaking communities of the Eastern Carpathians, with particular emphasis on the folk taxon *bergőburján*. Specifically, we sought to: (i) document local names, beliefs and uses associated with orchids; (ii) evaluate how knowledge varies among regions and demographic groups; and (iii) assess the extent to which historical records of orchid use and nomenclature persist in present-day traditional knowledge.

As our study was an exploratory ethnobotanical work based on non-random interview data, we did not formulate formal confirmatory hypotheses. Nevertheless, based on previous ethnobiological studies in the region and on the historical record of *bergőburján* from the former Csík County, we expected three main patterns. First, knowledge of *bergőburján* and recognition of orchid species would be more frequent in Gyimes than in the neighbouring Csík settlements. Second, older respondents would possess more extensive orchid-related knowledge than younger respondents, reflecting the intergenerational erosion of TEK. Third, *bergőburján*-related knowledge would persist mainly as remembered vernacular names, narratives and reported former uses, rather than as evidence of current practical use.

## 2. Materials and Methods

### 2.1. Study Area

**Figure 2:**
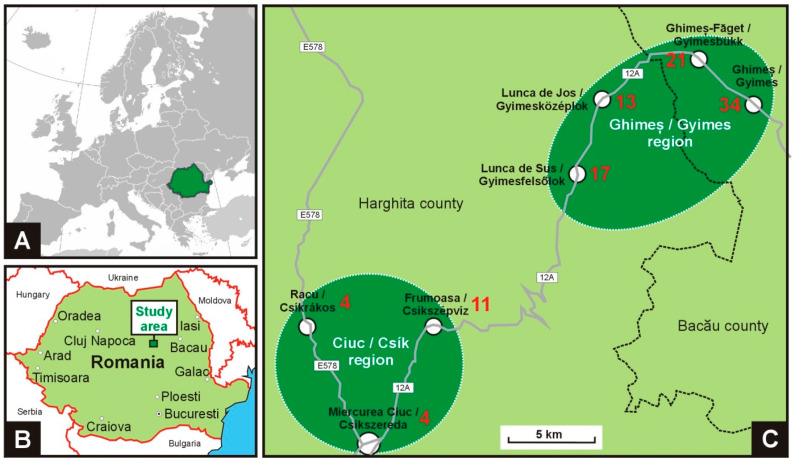
Map of the study area. (**A**) Location of Romania in Europe. (**B**) Location of the study area in Romania. (**C**) Sampled settlements in the Csík and Gyimes regions. The names of settlements and regions are given in Romanian followed by Hungarian, with the two forms separated by a slash. White circles mark the sampled settlements, and red numbers indicate the number of interviews conducted in each settlement. Scale bar on panel (**C**) is shown for spatial reference.

**Figure 3:**
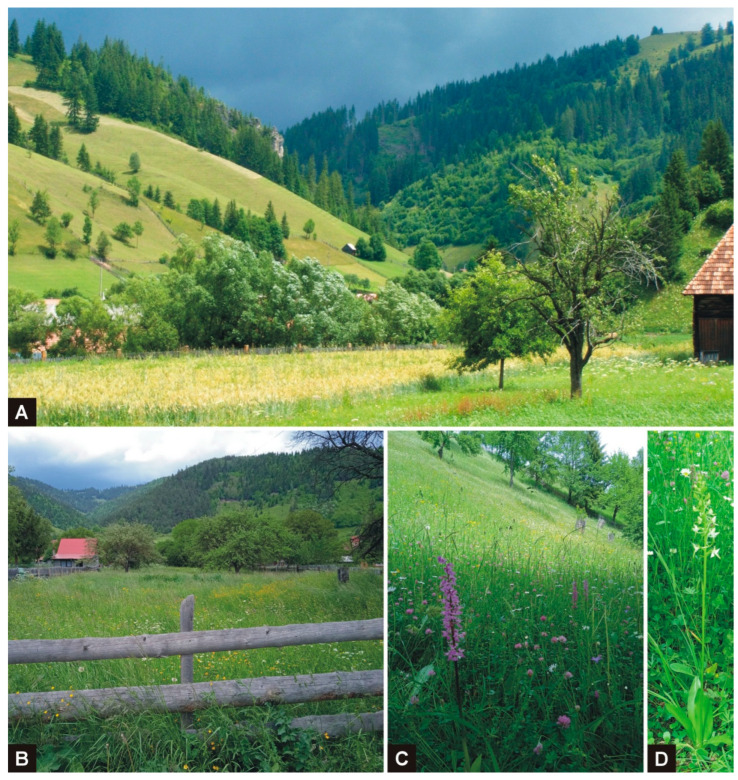
Typical landscape and orchid habitat in the Gyimes region. (**A**) Hay meadows and forests near Lunca de Jos/Gyimesközéplok. (**B**) Hay meadow of a key informant near Ghimeș/Gyimes. (**C**,**D**) Hay meadow of an informant with *Orchis mascula* subsp. *speciosa* and *Platanthera bifolia*, respectively, near Ghimeș/Gyimes. Credit: D.G. (**B**–**D**), A.M.V. (**A**).

The study area is located in the Eastern Carpathians, Romania (Figure 2), a mountainous landscape lying between 660 and 1500 m above sea level. The villages are situated in the valleys, with surrounding steep slopes covered by a mosaic of spruce (*Picea abies*) forests and hay meadows (Figure 3) [33,34,35]. The area is characterised by species-rich, regularly mowed semi-natural grasslands [34], which serve as habitats for numerous orchid species (e.g., *Anacamptis morio* (L.) R.M.Bateman, Pridgeon & M.W.Chase, *Dactylorhiza majalis* (Rchb.) P.F.Hunt & Summerh., *D. sambucina* (L.) Soó, *Gymnadenia conopsea* (L.) R.Br., *Nigritella rubra* K. Richt., *Orchis mascula* subsp. *speciosa* (Mutel) Hegi, *Platanthera bifolia* (L.) Rich., *Traunsteinera globosa* (L.) Rchb.), many of which occur in very high abundance [31]. The region is also well known for the exceptional persistence of TEK and traditional land-use practices among the local Hungarian-speaking Csángó communities [31,33,34,35].

The climate of the region is cool montane, with long winters, relatively short growing seasons and high precipitation compared with lowland areas of the Carpathian Basin. Mean annual temperature is generally around 5–7 °C, while annual precipitation commonly ranges between approximately 700 and 900 mm, depending on elevation and local topography [37]. These climatic conditions, together with long-term low-intensity mowing and grazing, contribute to the persistence of species-rich hay meadows and orchid habitats. Parts of the studied landscape and surrounding hay meadow areas overlap with, or are situated near, protected areas and Natura 2000 sites designated for the conservation of mountain grasslands and associated biodiversity.

### 2.2. Data Collection

A total of 104 semi-structured interviews [38] were conducted in Hungarian between 3 and 20 June and 6 and 18 October 2023 in seven settlements of the Csík and Gyimes regions. In the Csík region, interviews were conducted in Racu/Csíkrákos (Harghita County), Frumoasa/Csíkszépvíz (Harghita County), and Miercurea Ciuc/Csíkszereda (Harghita County). In the Gyimes region, interviews were conducted in Ghimeș/Gyimes (Bacău County), Ghimeș-Făget/Gyimesbükk (Bacău County), Lunca de Sus/Gyimesfelsőlok (Harghita County), and Lunca de Jos/Gyimesközéplok (Harghita County) (Figure 2).

Informants were not selected randomly. Recruitment combined opportunistic field-based sampling, local expert contacts and snowball sampling. During fieldwork, we approached people encountered in the villages, for example, in streets, courtyards, gardens, hay meadows and other everyday local settings, and attempted to interview all individuals who were willing to participate. In addition, two potentially knowledgeable informants were suggested to us by Ödön Balázs (Székelyudvarhely), who was familiar with the region and local communities. Snowball sampling was also applied: when interviewees recommended other people who might have relevant knowledge of local plant names, orchids, traditional land use or *bergőburján*, we attempted to contact and interview them as well.

The duration of the interviews varied between approximately 30 and 60 minutes. Audio recording was not used to maintain an informal interview situation and to minimise potential discomfort among respondents, especially elderly informants, but detailed notes were taken during the interviews and written down directly on the pre-printed data sheets. To provide a standardised framework for note-taking across interviews and fieldwork periods and to improve consistency, the same pre-printed data sheet and the same set of guiding questions were used during all interviews. Notes were written down directly during the conversations, and unclear or ambiguous answers were clarified immediately with the respondent whenever possible. Neither separate post-interview verification nor formal inter-observer reliability test was conducted. Responses that remained missing, uncertain or ambiguous after clarification during the interview were not inferred by the authors and were excluded from the relevant quantitative summaries and statistical analyses. Percentages were calculated using the number of valid responses for each question or category.

The unequal number of interviews between Gyimes (*n* = 85) and Csík (*n* = 19) reflects the primary focus of the study, rather than population size or an attempt to obtain balanced regional samples. *Bergőburján*-related knowledge has been historically and ethnographically most closely documented from the Gyimes region and the former Csík County, whereas the Csík settlements included here served mainly as neighbouring comparative sites. Therefore, the regional comparisons should not be interpreted as estimates derived from a balanced or random regional sample, but as exploratory comparisons indicating broad differences in the occurrence of *bergőburján*-related knowledge between the main study area and nearby settlements.

The first part of the interview focused on the socio-demographic characteristics of respondents (age, gender, occupation). Of the 104 interviewed informants, 64 were female, and 40 were male. The youngest interviewee was 18 years old, while the oldest was 87 years old (median ± SD: 55.5 ± 18.8 years). Detailed demographics of the interviewed informants are provided in Table 2. For some statistical analyses, respondents were classified into two age groups using the median age of the sample (56 years) as the threshold.

### 2.3. Photographic Recognition Task and Bergőburján Questionnaire

**Figure 4:**
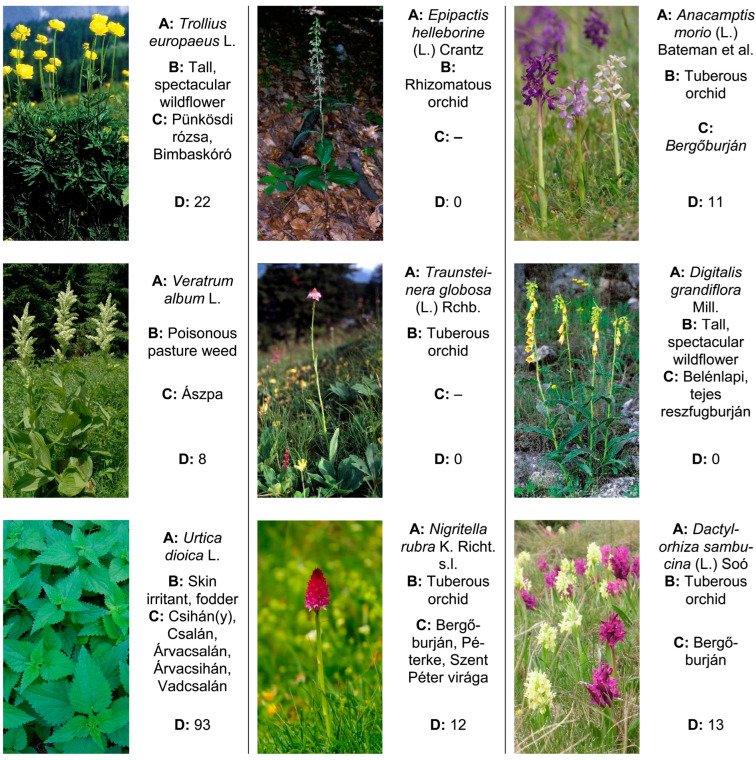
Plant species used in the photographic recognition task, all shown in flowering condition, with their scientific name (A), salience categories (B), vernacular names (C), and the number of informants who named each plant (D). An en dash (–) after C indicates that no vernacular name was found in the literature or recorded in the present study.

The second part of the interview aimed to elicit the interviewees’ plant-related knowledge. Colour-printed photographs of nine herbaceous plant species occurring in the study area (four tuberous orchids, one rhizomatous orchid, and four non-orchid species of significant saliency, see Figure 4) were presented, with the following question posed: “Do you recognise the plant depicted in these images?” All photographs were printed in colour at comparable size and resolution, and images of similar visual quality were selected to minimise differences in recognisability caused by photograph quality rather than by respondent knowledge. Photographs were selected to represent the appearance and condition of species most likely to be encountered by local inhabitants during the flowering period. Species were selected to represent both orchids and highly salient non-orchid plants commonly encountered in the study area, thereby allowing comparison of orchid recognition with recognition of culturally or ecologically prominent plant species. Orchid species were selected to include taxa previously reported to be associated with the folk taxon *bergőburján* in the region [30,31]. This task was used as a standardised elicitation method to compare recognition and naming of the selected species among respondents. It was not intended as a formal test of botanical identification accuracy or as a direct measure of field identification skills. The set of nine species was not intended to provide a comprehensive measure of overall plant knowledge, but it was used as a focused elicitation tool to assess recognition of selected orchids and to compare this recognition with that of several highly salient non-orchid plants from the same landscape. Species selection was informed by previous botanical and ethnobiological studies in the region, but no formal pilot study was conducted.

Finally, the following 11 questions were asked regarding the local knowledge of *bergőburján* and related information on its use and collection:

− Have you ever heard of *bergőburján*?
− What is *bergőburján*?
− Who told you about it?
− What is it used for?
− When was it last used?
− How was it used?
− Who consumed it?
− Who collected it?
− When was it collected?
− Was it collected for commercial purposes?
− Do you know anything else about *bergőburján*?

The questionnaire was developed based on the aims of the study, previous ethnobiological field experience in the region, and the earlier literature on *bergőburján* and local plant knowledge. The same set of guiding questions was used in all interviews, and all interviews were conducted in Hungarian, the shared language of the interviewers and respondents. The questionnaire was not formally validated, and no structured pilot study was conducted. The questions were kept simple and open-ended, and unclear answers were clarified during the interviews whenever possible.

Qualitative responses were processed descriptively. We did not conduct a formal thematic analysis. Answers recorded on the field data sheets were grouped into content-based categories relevant to the aims of the study. These included, for example, knowledge of the name only, ecological or morphological information, reported use, source of knowledge, collection time, user group, and joking or narrative associations. Categories were developed during data processing by repeated reading of the field notes and were used to summarise recurring response types rather than to produce an exhaustive qualitative coding framework.

Local Hungarian expressions quoted in the manuscript were translated into English by the authors and checked for consistency by the Hungarian-speaking co-authors. The translations were kept as literal as possible, while also preserving the contextual meaning of idiomatic expressions.

### 2.4. Statistical Analysis

To compare how many species were named by interviewees across two regions (Csík vs. Gyimes), between age groups (18–56 years vs. 57–87 years), between those who had heard of *bergőburján* in Gyimes, and between those who knew that *bergőburján* was used as an aphrodisiac, the Mann–Whitney test was applied. The median-based age grouping was used only for descriptive and exploratory group comparisons, allowing broad differences between younger and older respondents to be presented in a simple and easily interpretable form.

Similarly, the Mann–Whitney test was used to assess age differences between groups in Gyimes based on whether they knew about *bergőburján*, and whether they were aware of its aphrodisiac use. To assess gender differences in the knowledge of interviewees about *bergőburján* in Gyimes, an χ^2^-test was used.

Finally, Poisson generalized linear models (GLMs) were fitted to investigate the effects of multiple predictors on the number of named species. Age, gender, region, occupation, the knowledge of *bergőburján* (binary coded), and the knowledge of its aphrodisiac use (binary coded) were initially considered as explanatory variables. Age was included as a continuous predictor to retain the full information contained in the variable and to assess whether species recognition changed gradually with increasing age while accounting for the effects of other predictors. Overdispersion was assessed for the candidate Poisson GLMs and found to be negligible (dispersion ratio: 0.44–0.70), supporting the use of a Poisson error distribution. Because occupation was represented by a multi-level categorical predictor and was of specific interest, we first evaluated a model including this predictor to assess occupational differences. We then fitted a second set of models without occupation to evaluate the effects of the remaining predictors independently of this multi-level factor. The full models were then simplified by sequentially removing predictors to obtain the most parsimonious model according to Akaike’s Information Criterion (AIC). Residual diagnostics revealed no evidence of systematic deviations from model assumptions. As a verification step, we additionally compared all possible predictor combinations, which identified the same best-supported model.

All statistical analyses were conducted in R v4.5.3 [39], including the ’glmmTMB’ package [40,41] for Poisson GLM, the ‘DHARMa’ [42] and ‘performance’ [43] packages for model diagnostics. Effect sizes and 95% confidence intervals were reported as rank-biserial correlations for the Mann–Whitney tests, Cramér’s V for χ^2^-test, using the ‘effectsize’ package [44], and incidence rate ratios obtained by exponentiating the Poisson GLM coefficients.

## 3. Results

### 3.1. Knowledge of Bergőburján

While in the Csík region only one respondent was familiar with the term *bergőburján* (1/19; 5.3%), in Gyimes 54 informants knew it (54/85; 63.5%). Because the regional sample sizes were unequal and respondents were not selected randomly, this contrast should be interpreted as an exploratory indication of a broad regional difference rather than as a representative population-level estimate. At the settlement level, familiarity with *bergőburján* ranged from 58.8% to 75.0% among the sampled Gyimes settlements, while only one respondent from the Csík settlements reported knowing the term.

One-third of the respondents familiar with *bergőburján* knew only the name. A further 14.8% possessed TEK (Figure 4A), including information on its occurrence, morphology and ecology. For example, its location *(‘Tarhavason nő’/It grows on Tarhavas*), characteristic appearance (*‘Pityókája van’/It has a ‘pityóka’* (potato, actually: tuber); *‘Kicsi, sárga és piros’/Small, yellow, and red*; *‘Fehér és piros virágú, nem illatos’/It has white and red flowers, not fragrant*; *‘Tenyér alakú a gyökere’/Its root is palm-shaped*) was highlighted. Three independent respondents mentioned that wild boars eat its tubers. Eleven informants associated the plant with jokes and banter (e.g., *‘Bergőburjánt a férfinak!’/Bergőburján to the man*; *‘Igyál bergőburjánt!’/Drink bergőburján*; *‘Egyéé bergőburjánt, hogy bezsegjen a véred!’/Eat bergőburján to make your blood boil*; *‘Bergőburjánt ettéé? Mi van veled?’/’Did you eat bergőburján? What’s wrong with you?’*). However, most of them (8 informants) claimed to know nothing else about the plant.

Among the tuberous orchids, three species were mentioned in Gyimes by a total of 23 informants as *bergőburján* or *‘serkentőfű’* (*“stimulating herb”*) for *Dactylorhiza sambucina* and *D. majalis*, and as *‘Szent Péter virág’* (*‘Saint Peter’s flower’*, one time), *‘Péterke’* (two times), and *‘Péterke virág’* (*‘Péterke flower’*, one time) for *Nigritella rubra*. In addition, the tuberous orchid *Traunsteinera globosa* and the rhizomatous orchid *Epipactis helleborine* were not identified as *bergőburján* by any of the respondents. Among informants who named at least one orchid from the photographic set, the mean number of named orchid species was 1.61 ± 0.50, while the mean number of named species among all nine photographed plants was 3.04 ± 1.07. These values describe recognition and naming within the standardised photographic task and should not be interpreted as direct measures of field identification accuracy.

### 3.2. Traditional Uses of Bergőburján

**Figure 5:**
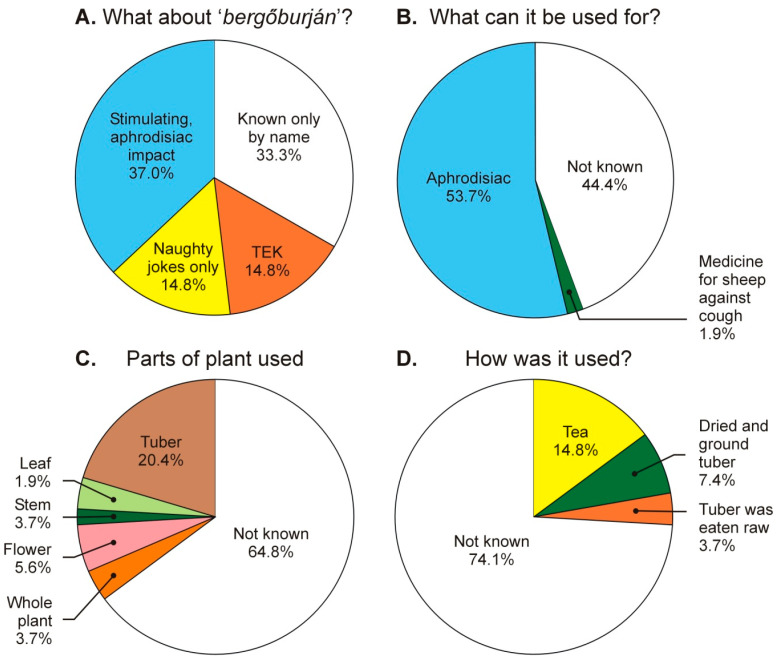
Recent (in the year of 2023) knowledge of orchids in the Gyimes region among the 54 informants who knew the term *bergőburján*. Percentages are calculated using these 54 informants as the denominator unless otherwise indicated.

Among respondents who were familiar with *bergőburján* (*n* = 54), only one informant (1.9%) mentioned that it was given to sheep against coughing and shedding, while 29 informants (53.7%) stated that it had been used for its aphrodisiac or stimulant effects (Figure 5B). Most of these mentions referred to remembered or reported former uses rather than to current practice or direct personal experience. Informants often framed this knowledge as something heard from older community members or known from local narratives. Eight informants mentioned that it was given to cattle and sheep, six informants to both livestock and humans, and 19 informants solely to humans. Among livestock, it was given exclusively to females (cows and ewes) when they had not become pregnant or when they were avoiding males. Among humans, both genders were mentioned as users. The most frequently used part (20.4%) was the tuber (Figure 5C), which was also consumed raw or used as tea for cows and humans. According to four key informants, it was also used dried and ground (Figure 5D). It was also given to maidens by bachelors without informing them. When girls were weaving yarn, they needed saliva for the task, so they chewed resin from spruce (*‘szurok’*), on which the boys secretly sprinkled the flour of dried tubers to make the girl fall in love. One informant mentioned they also secretly added it to each other’s drinks.

Among respondents familiar with *bergőburján* (*n* = 54), 23 informants (42.6%) did not know from whom they had heard about it; 15 (27.8%) said it was from “elders”, five (9.3%) from grandparents, four (7.4%) from parents, and seven (13.0%) from friends. This pattern indicates that much of the information recorded here represents socially transmitted and recollected knowledge rather than direct personal experience. The timing of its collection was reported by 18 respondents, most (72%) of whom stated it was in the spring, with five (28%) mentioning the flowering stage (e.g., *‘Májusban, amikor a virág teljében volt’/In May, when the flower was in full bloom*; *‘Virágzáskor, amikor látszott, hogy mi az’/When it was flowering, and you could see what it was*).

Most of the respondents (75.9%) did not know who used to collect *bergőburján*, while 11 respondents (20.4%) stated that everyone collected it for their own use, and only two respondents (3.7%) mentioned collecting it for commercial purposes in the past. The use of *bergőburján* is becoming obsolete, with most respondents (64.8%) unable to say when it was last used. 25.9% said it was more than 40 years ago (up to 60–70 years ago), 7.4% said 10–30 years ago, and only one informant mentioned recent use.

### 3.3. Vernacular Names of Orchids

Several vernacular names associated with tuberous orchids were recorded during the interviews (Figure 4). The most frequent and culturally most salient name was *bergőburján*, which functioned as a broader folk taxon and was applied collectively to several tuberous orchid species. In Gyimes, three tuberous orchid species were mentioned by a total of 23 informants as *bergőburján* or *serkentőfű* (*“stimulating herb”*), the latter name being recorded for *Dactylorhiza sambucina* and *D. majalis*. Additional names were rare and taxon-specific. *Nigritella rubra* was referred to as *Szent Péter virág* (*“Saint Peter’s flower”*) by one informant, *Péterke* by two informants, and *Péterke virág* (*“Péterke flower”*) by one informant. These low-frequency names indicate that vernacular orchid nomenclature was not uniform but varied among informants and taxa.

### 3.4. Recognition of Orchids and Other Plants

Among the plants shown in photographs, *Urtica dioica* was the most frequently recognised species (89.4%). Recognition differed between the two regions (Table 3). Informants from Gyimes named an average of 1.65 ± 1.05 species, whereas those from Csík named 0.95 ± 0.71 species. The regional, gender- and age-related patterns are summarised in Table 4, which allows comparison of the mean number of named species among the main respondent groups.

Interviewees in Gyimes named statistically significantly more species (W = 1090, *p* = 0.006, r_rb_ = 0.35 [0.08, 0.57]) than in Csík. Similarly, older interviewees named more species (W = 1685.5, *p* = 0.010, r_rb_ = 0.25 [0.04, 0.45]) than younger interviewees. In Gyimes, those who had heard of *bergőburján* (W = 1092, *p* = 0.006, r_rb_ = 0.30 [0.06, 0.52]) or knew that it was used as an aphrodisiac (W = 1129.5, *p* < 0.001, r_rb_ = 0.39 [0.15, 0.59]) named significantly more species. Knowledge of *bergőburján* was similar between genders in Gyimes (χ^2^ = 0, *p* = 1, Cramér’s V = 0).

**Table 5:** Summary of the two most parsimonious Poisson GLMs investigating the relationships between the number of named species and its predictors. Model 1 was used to evaluate occupational differences in species recognition, whereas Model 2 examined the effects of the remaining predictors after excluding occupation. For categorical predictors, the base of the comparison is shown in parentheses, and the compared groups with estimated values are displayed in subsequent rows. The Akaike’s Information Criterion (AIC) value, the difference (ΔAIC) compared to the corresponding full model, and McFadden’s pseudo-R^2^ value are shown at the end of the table. Significant *p* values (≤0.05) are marked (*).

Predictor	Model 1			Model 2		
	β	IRR [95% CI]	*p*	β	IRR [95% CI]	*p*
(Intercept)	0.616	1.852 [1.357, 2.527]	<0.001 *		0.583 [0.306, 1.112]	
Occupation (Farmer)						
Housewife	−0.464	0.629 [0.339, 1.166]	0.141			
Intellectual	−0.598	0.55 [0.252, 1.202]	0.134			
Manual worker	−0.604	0.547 [0.366, 0.816]	0.003 *			
Retired	−0.249	0.78 [0.485, 1.253]	0.304			
Student	−0.462	0.63 [0.282, 1.406]	0.259			
Knew as an aphrodisiac	0.361	1.435 [1.024, 2.013]	0.036 *	0.335	1.398 [1, 1.955]	0.050 *
Age				0.009	1.009 [1, 1.018]	0.042 *
Region (Csík)						
Gyimes				0.423	1.527 [0.922, 2.529]	0.100
AIC	287.547			284.688		
ΔAIC	4.083			3.585		
McFadden’s R^2^	0.065			0.054		

### 3.5. Predictors of Species Knowledge

Based on the results of the Poisson GLMs, knowledge of the aphrodisiac use of *bergőburján* was a significant predictor of species recognition, and manual workers named significantly fewer species than farmers (Table 5). Since occupation was a multi-level factor, we excluded it from a separate model to evaluate the effects of the other predictors. Then, age exerted a statistically significant positive effect besides the knowledge of aphrodisiac use of orchids (Table 5), which is congruent with the findings detailed in the previous subsections.

## 4. Discussion

### 4.1. Persistence of Traditional Knowledge Related to Bergőburján

The assumptions regarding the aphrodisiac properties of the testicle-shaped tubers of terrestrial orchids stem from the ancient Doctrine of Signatures. Tuberous orchids are still harvested and consumed in several parts of the Eastern Mediterranean and Western Asia, particularly in the form of *salep* beverages and ice cream [5,6,7,8,9,10,11,12,13,14,15,16]. However, their use as aphrodisiacs appears to have largely disappeared from most European regions. Historical Hungarian botanical, linguistic and ethnomedical sources, however, provide an important comparative background for interpreting the persistence of such beliefs in present-day local knowledge. Veszelszki [25] described contrasting effects attributed to fresh and withered orchid tubers, while Diószegi [26] referred to orchids as “arousing love”. Berde [27] mentioned their use against impotence. Wagner [28] recorded the vernacular name *bergőburján* and reported that orchid tubers were administered to both humans and livestock because they were believed to stimulate sexual activity. This semantic association was also reflected in a contemporary explanation: “*Akivel megetetik, megitatják, jobban berreg”/*“*Whoever is made to eat or drink it will ‘berreg’ more intensely”*, directly linking the plant name to sexual arousal or mating behaviour. The continuity with Wagner’s nineteenth-century documentation is further suggested by recent expressions such as “*Megbolondította a nőket*”/“*It made women lose their senses*”, which closely echoes the older idea that the appropriate tubers would “*elfogja őket a bolondság*”/“*they become foolish with desire*”.

**Figure 6:**
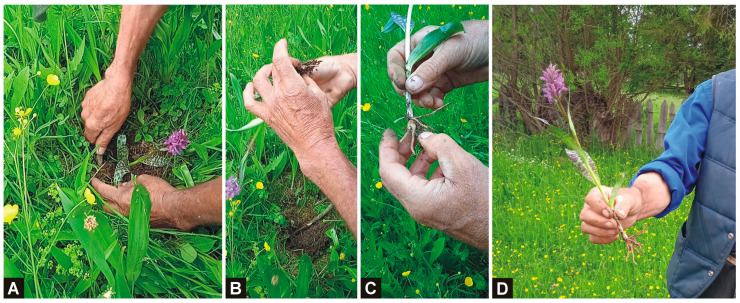
Field-based orchid recognition by a key informant in Gyimes. The informant identified and collected *Dactylorhiza majalis* on his own hay meadow near Ghimeș/Gyimes: (**A**) digging up the plant he had found using a pocketknife; (**B**) remowing adhering soil from the tuber; (**C**) displaying the tuber; and (**D**) presenting the entire excavated plant as ‘serkentőfű’ (‘stimulating herb’). The photographs have been cropped so that the informant is not recognisable. Credit: T.H.

One of the key findings of the present study is that contemporary *bergőburján*-related knowledge shows clear similarities with historical records from the same region, especially Wagner’s nineteenth-century documentation. This finding aligns well with recent work on *Cicuta virosa* in Hungary, where historical digital archives revealed not only former occurrence records but also a rich body of vernacular names, poisoning narratives, and ethnomedicinal and ethnoveterinary knowledge [36]. In both cases, older Hungarian sources provide more than antiquarian detail: they serve as baselines against which the persistence, transformation, or erosion of local plant knowledge can be evaluated. Approximately one-third of the informants familiar with *bergőburján* still associated it with aphrodisiac effects, and a substantial proportion retained information on its morphology, habitat, collection and use. Furthermore, some elderly informants were still able to identify orchid species from photographs and locate them in their natural habitats (Figure 6). These observations suggest that elements of orchid-related traditional knowledge documented in historical sources have not disappeared completely but remain recognisable within parts of the local population.

Direct comparison of the mean number of named orchid species with other ethnobotanical studies is difficult, because studies differ substantially in the number and type of species presented, interview design, local floras and cultural contexts. We therefore use this value primarily as an internal measure for comparing recognition among respondent groups within the present study.

The striking contrast between the neighbouring regions of Gyimes and Csík further emphasises the cultural significance of this phenomenon. While *bergőburján* and its associated beliefs remain relatively well known in Gyimes, they are almost entirely absent from Csík. However, this regional contrast should be interpreted cautiously because of the unequal sample sizes and non-random sampling design. In addition to the historical and cultural context of the Gyimes Csángó communities, differences in local land-use practices, livelihood structure, education, migration history and everyday exposure to hay meadows and livestock husbandry may also have contributed to the observed pattern. This pattern is consistent with previous ethnobiological studies from the region [45,46,47,48]. These studies show that the Csángó communities of Gyimes have preserved numerous archaic elements of TEK, folk culture and land-use traditions owing to their relative geographical and cultural isolation. The persistence of *bergőburján*-related knowledge may therefore represent another example of the survival of local ecological and cultural traditions in the Eastern Carpathians.

A practical explanation for the use of orchid tubers in ethnoveterinary practices was provided by two of our key informants from Gyimesközéplok. In the traditional local farming system, the timing of calving, lambing and subsequent milk production was of considerable economic importance. Animals were therefore sometimes “stimulated” to reproduce earlier to ensure that milk production coincided with the seasonal cycle of hay-making and cheese production. As an informant explained, “*Meg volt az ideje, mikor kell a tej, nem volt mindegy, hogy mikor ellett az állat”/”There was a specific time when milk was needed; it mattered when the animal gave birth”*. A second informant gave a similar explanation, noting that “*Amikor a földeken kellett dolgozni, már nem volt idő a sajtkészítésre*”/“*When people had to work in the cropfields, there was no time for cheese making*”. These mentions suggest that the reputed stimulating effect of *bergőburján* was embedded not only in symbolic ideas of fertility. It was also connected to the practical temporal organisation of traditional livestock husbandry. These accounts should be interpreted as local retrospective explanations provided by key informants, rather than as independently verified historical evidence for the organisation of livestock husbandry.

In addition, the dual human and veterinary interpretation was concisely summarised by one informant: “*Van aki a tehénnek adta, amelyik nem folyatott, van aki az asszonynak*” (“*Some gave it to the cow that did not come into heat, others gave it to the woman*”).

### 4.2. Folk Taxonomy and Vernacular Nomenclature

The vernacular nomenclature documented in this study provides valuable insights into local folk taxonomy. The name *bergőburján* functions as a collective folk taxon encompassing several tuberous orchid species rather than a single botanical entity. Similar collective folk taxa are common features of traditional classification systems and frequently group together biologically distinct species that share salient morphological, ecological or cultural characteristics [49]. Comparable use-based groupings are also known from orchid ethnobotany in regions where several tuberous orchid species are collected or used for similar purposes, especially in relation to *salep*, medicine or symbolic interpretations of tubers [5,6,7,8,9,10,11,12,13,14,15,16,18,19,20,21]. In the present case, the folk category *bergőburján* appears to be structured primarily by shared underground morphology and presumed stimulating effects, while habitat association may have reinforced recognition because several of the relevant species occur in the same hay meadow and pasture landscapes.

The same vernacular name was applied to different species of genera such as *Dactylorhiza*, *Nigritella* and *Platanthera.* This suggests that local classification is based less on taxonomic distinctions recognised by professional botanists than on shared underground morphology, perceived biological effects and cultural significance. This observation is consistent with the view that folk classification systems often reflect culturally meaningful relationships rather than the taxonomic criteria used in scientific botany [50].

The recorded names *serkentőfű* (“stimulating herb”), *Péterke, Péterke virág, Szent Péter virága* (Saint Peter’s flower) and the historically documented *vitézvér* (“valiant blood”) further illustrate the cultural richness of orchid-related nomenclature in the region. The name *serkentőfű* directly reflects the presumed physiological (aphrodisiac) effects of orchid tubers. By contrast, the names associated with *Nigritella rubra* appear to be linked to local symbolic and religious traditions. Such utilitarian and symbolic naming systems are characteristic elements of TEK and often encode culturally important information regarding species and their uses [45]. One distinctive element of Wagner’s historical documentation [28] was the gender-differentiated interpretation of tuber morphology, according to which rounded tubers were associated with men and fingered tubers with women. Although some contemporary informants retained morphological knowledge of orchid tubers, including references to their rounded or palm-shaped form, we did not record a clear present-day distinction corresponding to this gendered classification. This suggests that the specific component of the historical knowledge complex has not clearly persisted in contemporary local knowledge, even though the broader association between *bergőburján*, sexuality and stimulation remains recognisable.

Importantly, these vernacular names should not be regarded merely as linguistic curiosities. They form part of a broader knowledge system that includes species recognition, habitat knowledge, collection practices, beliefs and oral traditions. The persistence of such names therefore reflects the survival of a much more complex body of TEK.

### 4.3. Erosion of TEK

Despite the persistence of orchid-related knowledge among some older informants, our cross-sectional data indicate marked age-related differences that are consistent with an ongoing erosion of this knowledge system. Older respondents possessed significantly greater knowledge of orchids and were more likely to be familiar with *bergőburján* than younger participants. However, these age-related differences should not be interpreted as the effect of age alone, because they may also reflect differences in education, occupation, migration history, lifestyle, involvement in livestock husbandry and hay meadow management, and opportunities for everyday interaction with orchid habitats. Likewise, knowledge of orchid uses, vernacular names and collection practices was concentrated among elderly informants (Figure 5).

The observed pattern closely supports previous findings from the Eastern Carpathians. Biró et al. [35] demonstrated that TEK related to threatened plant species was unevenly distributed among community members and strongly associated with practical experience and direct interaction with species and habitats. Our results suggest that a similar process affects orchid-related knowledge in Gyimes. A substantial proportion of older inhabitants still retains information on orchid recognition, habitat preferences and former uses. However, this knowledge is becoming progressively detached from everyday practice. As a result, it is increasingly vulnerable to disappearance. Beyond their statistical significance, these group differences also have practical and cultural importance. They indicate that orchid-related knowledge is not evenly distributed within the local population but is concentrated among older informants and among respondents from Gyimes, where traditional hay meadow management, livestock husbandry and everyday interaction with orchid habitats have remained more visible. Thus, the observed differences should be interpreted not only as demographic patterns, but also as indicators of the social and cultural structuring of plant knowledge.

The persistence of *bergőburján* in sexual jokes and banter also indicates that TEK may persist in transformed social registers after practical use has declined. For several informants, the name was not associated with detailed botanical knowledge or direct experience of use, but with humorous expressions and socially transmitted narratives concerning sexuality and stimulation. This suggests that *bergőburján*-related knowledge has partly shifted from practical ethnobotanical knowledge toward symbolic, narrative and humorous forms of cultural memory. Such persistence of a vernacular name and its associations may represent an intermediate stage in TEK erosion, in which practical application has largely disappeared, but culturally meaningful references continue to circulate within the community. From a contemporary ethnobiological and ethical perspective, such beliefs raise questions about the social and ethical dimensions of traditional aphrodisiac use.

Importantly, the decline observed in our study does not necessarily represent a complete loss of knowledge. It may reflect a transformation of the socio-ecological context in which knowledge was formerly generated, transmitted and applied. As argued by Biró et al. [35], the apparent absence of knowledge among younger generations may reflect reduced opportunities to acquire practical experience through direct interaction with species and habitats. Similar processes have been documented worldwide, where rapid socio-economic changes and modernisation have contributed to declining intergenerational transmission of local ecological knowledge [51].

The near disappearance of practical orchid use provides additional support for this process. Most respondents were unable to specify when *bergőburján* had last been used, and only a single informant reported recent use. According to one respondent, “*már kevés az állat, ezért nem használják”/”there are few livestock left, so it is not used*”. This concise statement may summarise the underlying mechanism of knowledge erosion effectively. As traditional livestock husbandry declines, the practical need for orchid-related knowledge disappears, and with it the opportunities for its transmission. Such drivers have repeatedly been identified as major causes of traditional knowledge erosion in rural socio-ecological systems [46]. The occupational difference observed here may also reflect variation in everyday exposure to the landscape. Farmers named more species than manual workers, possibly because farming, hay-making, grazing and livestock care provide repeated opportunities for direct interaction with grassland plants and orchid habitats. By contrast, manual workers may have fewer regular occasions to acquire or maintain such plant-related knowledge through everyday practice. However, occupation was recorded only as a broad socio-demographic category, and the non-random sampling design does not allow causal interpretation. This result should therefore be regarded as an exploratory pattern consistent with the broader link between practical land-use experience and TEK.

### 4.4. Cultural and Conservation Implications

Although orchids are globally threatened [47], and many studies have highlighted the conservation risks associated with harvesting tuberous species [5,6,9,10,11,12,14,15,16], our interview data do not suggest that tuber collection currently represents a major threat in Gyimes. Extensive and locally abundant populations of several tuberous orchid species still occur in the highly species-rich semi-natural grasslands of the region, particularly in traditionally managed hay meadows, despite the former collection and use of tubers. Moreover, the interviews indicate a marked erosion of knowledge related to *bergőburján* and an almost complete decline of its practical use. This suggests that present-day collection pressure is probably low, although this was not assessed through direct field monitoring of harvesting intensity. Consequently, in the studied region, the principal conservation concern appears to be not overharvesting, but rather the gradual abandonment, intensification or transformation of the traditional land-use systems that historically maintained both orchid-rich grasslands and orchid-related TEK.

The conservation relevance of the documented knowledge is therefore linked less to the direct regulation of harvesting than to the maintenance of orchid-rich biocultural landscapes. The folk taxon *bergőburján* was mainly associated with tuberous orchids of species-rich semi-natural grasslands, including taxa such as *Anacamptis morio*, *Dactylorhiza majalis*, *D. sambucina*, *Gymnadenia conopsea*, *Nigritella rubra*, *Orchis mascula* subsp. *speciosa* and *Platanthera bifolia*. These species are characteristic elements of traditionally managed hay meadows and pastures, and their persistence depends largely on the continuation of low-intensity grassland management, especially regular mowing, moderate grazing and the avoidance of abandonment, intensification or afforestation. Local traditional knowledge may contribute to conservation by helping to identify culturally important orchid populations, supporting community-based habitat monitoring, strengthening local acceptance of grassland conservation measures and reinforcing the biocultural value of traditional hay meadow landscapes. In practical terms, this would require cooperation among local farmers and hay meadow owners, community members familiar with orchid populations, conservation practitioners, protected-area managers and local authorities. Such cooperation could support the continuation of low-intensity mowing and moderate grazing, prevent abandonment, intensification or afforestation of species-rich meadows, and integrate local knowledge into habitat monitoring and management decisions.

The concept of biocultural diversity emphasises the interconnected evolution of biological and cultural systems and recognises that losses in biodiversity are frequently accompanied by losses in cultural knowledge and practices [48]. The present study provides a clear example of this phenomenon. Orchid-related knowledge in Gyimes encompasses vernacular nomenclature, ecological observations, ethnoveterinary practices, beliefs concerning fertility and sexuality, and practical knowledge of species occurrence. Together, these elements form part of the region’s biocultural heritage. In this sense, the conservation of orchid-rich hay meadows in Gyimes should be understood not only as habitat protection, but also as the maintenance of a biocultural system in which species, local names, management practices and oral narratives are mutually connected. Ethnobotanical knowledge can therefore contribute to conservation by linking biological values with local meanings, community memory and everyday land-use practices.

The species-rich hay meadows of the Eastern Carpathians represent an outstanding example of a biocultural landscape. For centuries, traditional land use has contributed simultaneously to biodiversity conservation and the maintenance of TEK [31,33,34,52]. However, these systems are increasingly threatened by the decline of traditional livestock husbandry and haymaking practices, agricultural intensification, land abandonment, habitat fragmentation, afforestation, climate change, tourism pressure and by broader socio-economic changes affecting rural communities [31,34,52,53]. Similar relationships between traditional land use, biodiversity and knowledge transmission have been documented in numerous traditional agricultural landscapes worldwide [52].

The *bergőburján* tradition illustrates how biodiversity, language, TEK and land-use practices may become integrated within a single socio-ecological system. From this perspective, TEK should be regarded not merely as a record of past practices but also as a potential resource for understanding and managing biocultural landscapes [53,54]. Documenting such knowledge is therefore important not only from an ethnobotanical perspective, but may also contribute to conserving the biocultural heritage of traditional rural landscapes and strengthening local connections to biodiversity [55,56].

### 4.5. Methodological Limitations

Several methodological limitations of our study should be acknowledged. First, informants were not selected through random sampling. Recruitment combined opportunistic field-based sampling, local expert contacts and snowball sampling. Consequently, the sample should not be interpreted as statistically representative of the entire population of the studied settlements. The markedly unequal sample sizes from Gyimes and Csík further limit the strength of regional comparisons. Therefore, these results should be regarded as exploratory patterns rather than as representative estimates for the two regions. The study was designed primarily as an ethnobotanical documentation of local knowledge, with particular emphasis on the persistence, transformation and erosion of knowledge related to tuberous orchids and the folk taxon *bergőburján*.

Second, the interviews were not audio-recorded. Although the same pre-printed data sheets and guiding questions were used throughout the fieldwork, and notes were taken directly during the conversations, the absence of audio recordings may have limited the verbatim accuracy and completeness of some vernacular expressions and oral testimonies. No separate post-interview verification and no formal inter-observer reliability test were conducted. These limitations may have introduced some degree of interviewer-related bias in the recording and interpretation of qualitative responses. For this reason, only expressions and statements that were clearly recorded during fieldwork and could be interpreted unambiguously in their interview context were used in the manuscript.

Third, the qualitative categories used in the Results were descriptive and content-based and were not derived from a formal thematic analysis. They should therefore be interpreted as pragmatic summaries of recurring response types rather than as the outcome of a fully developed qualitative coding procedure. Similarly, the questionnaire was not formally validated through a structured pilot study. It should be regarded as a semi-structured ethnobotanical elicitation tool designed to document local names, uses and narratives, rather than as a formally validated instrument for measuring plant knowledge.

Fourth, the photographic recognition task also had inherent limitations. Although it provided a standardised stimulus across interviews, it did not reproduce all cues available during real-life plant recognition, such as habitat context, seasonality, plant size, smell, tactile experience or repeated direct interaction with plants. In addition, the October interviews were conducted outside the flowering period of most species included in the photographic recognition task, which may have reduced respondents’ immediate familiarity with the plants and influenced recognition performance. Therefore, the number of species named from photographs should be interpreted as a measure of recognition within the interview setting, not as a direct measure of field identification ability. Moreover, all respondents completed the same standardised recognition task based on nine selected plant species. Consequently, the results should not be interpreted as a measure of overall plant knowledge, since the task was designed specifically to assess recognition of selected orchid species and several other salient species occurring in the same landscape.

Finally, reported former uses of *bergőburján* should be interpreted with caution. In most cases, the interviews documented remembered or socially transmitted knowledge rather than directly observed practices. These accounts therefore provide evidence for the persistence of local narratives and remembered use categories, but they cannot be used to reconstruct the exact frequency, timing or continuity of actual historical use. The dataset was not designed as a comprehensive species–use–informant inventory; consequently, broader quantitative ethnobotanical indices or network analyses linking species, uses and informants were not applied. Such approaches would require a more systematic and exhaustive documentation of plant–use relationships across the local flora and could be usefully applied in future studies.

## Figures and Tables

**Table 1:** Original Hungarian sources and their English translations related to the aphrodisiac effect of orchids.

Source, Year of Publication	Original (Hungarian) Text	English Translation
Veszelszki [25] (p. 330) 1798	*a’ gyökere kettős tökforma, egyik fris, a’ másik pedig meg-hervadt forma: A’ kettős gyökérnek kettős a’ haszna: mert a’ frissel élünk, hogy a’ férjfiúság megerősödjön; a’ hervadttal pedig ad coercendam libidinem, hogy a’ bujaságot elóltsuk magunkban*	Its root is double, resembling testicles, one fresh, the other withered. The double root has double benefit: we use the fresh to strengthen male virility; and with the withered one, we give ad coercendam libidinem, to extinguish lust within ourselves.
Diószegi [26] (p. 312) 1813	*szerelemre gerjesztő*	arousing love
Wagner [28] (p. 142) 1899	*bergő burján: kosbor fajok (Orchisok). Két csoportot különböztetnek meg: az ujjas gumójú a fehérnépé, a gömbölyű gumós a legényeké. A megfelelő gumókat élvezve »elfogja őket a bolondság«. A marhának is adják, hogy megűződjék.*	Bergő burján: *Orchis* species. Two groups are distinguished: women have the fingered tubers, and bachelors have the rounded ones. Enjoying the right tubers »they become foolish with desire«. They are also given to cattle to make them receptive.
Berde [27] (p. 142) 1940	*impotencia ellen*	against erectile dysfunction

**Table 2:** Selected demographics of interviewed informants (*n* = 104).

	*n*	Percentage
Age (Years)		
18–19	6	5.8%
20–29	11	10.6%
30–39	16	15.4%
40–49	10	9.6%
50–59	20	19.2%
60–69	24	23.1%
70–79	9	8.7%
>80	8	7.7%
Gender		
Female	64	61.5%
Male	40	38.5%
Nature of occupation		
Manual worker	68	36.5%
Farmer	29	27.5%
Retired	16	15.4%
Housewife	9	8.7%
Intellectual	6	5.8%
Student	6	5.8%

**Table 3:** Comparison of the identification of plants shown in photographs during interviews in the two study regions. Names of tuberous orchids are highlighted in boldface.

Species	Region	Known by Name n (Percentage)	Not Known n (Percentage)
*Urtica dioica*	Ciuc/Csík	11 (57.9%)	8 (42.1%)
	Ghimeș/Gyimes	82 (96.5%)	3 (3.5%)
*Trollius europaeus*	Ciuc/Csík	3 (15.8%)	15 (84.2%)
	Ghimeș/Gyimes	19 (22.4%)	66 (77.6%)
***Dactylorhiza sambucina***	Ciuc/Csík	0 (0%)	19 (100%)
	Ghimeș/Gyimes	13 (15.3%)	72 (84.7%)
***Nigritella rubra***	Ciuc/Csík	0 (0%)	19 (100%)
	Ghimeș/Gyimes	12 (14.1%)	73 (85.9%)
***Anacamptis morio***	Ciuc/Csík	0 (0%)	19 (100%)
	Ghimeș/Gyimes	11 (12.9%)	73 (87.1%)
*Veratrum album*	Ciuc/Csík	0 (0%)	19 (100%)
	Ghimeș/Gyimes	8 (9.4%)	77 (90.6%)

**Table 4:** Number of plants named by different respondent groups. Values are shown as mean ± SD; effect sizes for the main statistical comparisons are reported in the text.

	Csík		Gyimes	
Gender	Male	Female	Male	Female
n	4	15	36	49
Mean ± SD	1.25 ± 0.96	0.87 ± 0.64	1.64 ± 1.15	1.65 ± 0.99
Age	18–56 years	57–84 years	18–56 years	57–87 years
n	10	9	44	41
Mean ± SD	0.9 ± 0.57	1.0 ± 0.87	1.32 ± 0.74	2.0 ± 1.22

## Data Availability

The datasets generated and analysed during the current study are not publicly available because they contain contextual information that could enable the indirect identification of informants from the small communities studied. De-identified data may be made available by the corresponding author upon reasonable request, subject to ethical and confidentiality considerations.

## References

[1] Pearn J. (2012). The doctrine of signatures, materia medica of orchids, and the contributions of doctor–orchidologists. Vesalius.

[2] Efferth T., Greten H.J. (2016). Doctrine of signatures—Mystic heritage or outdated relict from middle-aged phytotherapy. Med. Aromat. Plants.

[3] Schiff J.L., Schiff J.L. (2018). History of orchids. Rare and Exotic Orchids: Their Nature and Cultural Significance.

[4] Teoh E.S., Teoh E.S. (2019). An ancient fantasy: Salep as aphrodisiac. Orchids as Aphrodisiac, Medicine or Food.

[5] Sezik E., Şener B. (2002). Destruction and conservation of Turkish orchids. Biodiversity—Biomolecular Aspects of Biodiversity and Innovative Utilization.

[6] Sezik E. (2002). Turkish orchids and salep. Acta Pharm. Turc..

[7] Tamer E.C., Karaman B., Copur U.O. (2006). A traditional Turkish beverage: Salep. Food Rev. Int..

[8] Pieroni A., Rexhepi B., Nedelcheva A., Hajdari A., Mustafa B., Kolosova V., Ganfaglione K., Quave C.L. (2013). One century later: The folk botanical knowledge of the last remaining Albanians of the upper Reka Valley, Mount Korab, Western Macedonia. J. Ethnobiol. Ethnomed..

[9] Kreziou A., de Boer H., Gravendeel B. (2016). Harvesting of salep orchids in north-western Greece continues to threaten natural populations. Oryx.

[10] Charitonidou M., Stara K., Kougioumoutzis K., Halley J.M. (2019). Implications of salep collection for the conservation of the elder-flowered orchid (*Dactylorhiza sambucina*) in Epirus, Greece. J. Biol. Res.-Thessalon..

[11] Molnár V.A., Takács A., Mizsei E., Löki V., Barina Z., Sramkó G., Tökölyi J. (2017). Religious differences affect orchid diversity of Albanian graveyards. Pak. J. Bot..

[12] Molnár V.A., Nagy T., Löki V., Süveges K., Takács A., Bódis J., Tökölyi J. (2017). Turkish graveyards as refuges for orchids against tuber harvest. Ecol. Evol..

[13] Molnár V.A., Süveges K., Molnár Z., Löki V. (2017). Using local people’s traditional ecological knowledge in discovery of rare plants: A case study from Turkey. Acta Soc. Bot. Pol..

[14] Molnár V.A., Löki V., Verbeeck M., Süveges K. (2021). Orchids of Azerbaijani cemeteries. Plants.

[15] Ghorbani A., Gravendeel B., Naghibi F., de Boer H. (2014). Wild orchid tuber collection in Iran: A wake-up call for conservation. Biodivers. Conserv..

[16] Ghorbani A., Gravendeel B., Zarre S., de Boer H. (2014). Illegal wild collection and international trade of CITES-listed terrestrial orchid tubers in Iran. Traffic Bull..

[17] Bazzicalupo M., Calevo J., Smeriglio A., Cornara L. (2023). Traditional, therapeutic uses and phytochemistry of terrestrial European orchids and implications for conservation. Plants.

[18] Hossain M.M. (2011). Therapeutic orchids: Traditional uses and recent advances—An overview. Fitoterapia.

[19] Dénes A., Papp N., Babai D., Czúcz B., Molnár Z. (2012). Wild plants used for food by Hungarian ethnic groups living in the Carpathian Basin. Acta Soc. Bot. Pol..

[20] Hinsley A., de Boer H.J., Fay M.F., Gale S.W., Gardiner L.M., Gunasekara R.S., Veldman S. (2017). A review of the trade in orchids and its implications for conservation. Bot. J. Linn. Soc..

[21] Ticktin T., Charitonidou M., Douglas J., Halley J.M., Hernández-Apolinar M., Liu H., Mondragón D., Pérez-García E.A., Tremblay R.L., Phelps J. (2023). Wild orchids: A framework for identifying and improving sustainable harvest. Biol. Conserv..

[22] Forgó G. (1817). Rendkívül való szükség idején, a közönségesen szokásban lévő gabona fajokon kívül, miből készíthetni még kenyeret hazánkban, s mit találhatni még, a mivel olyankor táplálhassa magát a szegénység?. Tud. Gyűjt..

[23] Fäller J. (1943). Növényeink a Népies Gyógyászatban, Kuruzslásban és Babonában. Ph.D. Thesis.

[24] Augustin B., Jávorka S., Giovannini R., Rom P. (1948). Magyar Gyógynövények I–II.

[25] Veszelszki A. (1798). A’ Növevény-Plánták’ Országából-Való Erdei, és Mezei Gyűjtemény, Vagy-Is fa és Fűszeres Könyv.

[26] Diószegi S. (1813). Orvosi Füvész-Könyv, Mint a Magyar Füvész-Könyv Praktika Része.

[27] Berde K. (1940). A Magyar nép Dermatologiája. A Bőr és Betegségei Népünk Nyelvében, Hiedelmeiben és Szokásaiban.

[28] Wagner J. (1899). Növény- és állatnevek. Magy. Nyelvőr.

[29] Rácz J. (2000). Agármony és bakszakáll. Magy. Nyelvőr.

[30] Molnár Z., Babai D. (2009). Népi növényzetismeret Gyimesben I. Növénynevek, népi taxonómia, az egyéni és közösségi növényismeret. Bot. Közlem..

[31] Babai D., Molnár Á., Molnár Z. (2014). “Ahogy Gondozza, Úgy Veszi Hasznát”. Hagyományos Ökológiai Tudás és Gazdálkodás Gyimesben (Traditional Ecological Knowledge and Land Use in Gyimes, Eastern Carpathians).

[32] Rácz J. (2000). Here és orr a botanikában. Magy. Nyelvőr.

[33] Babai D., Molnár Z. (2013). Multidimensionality and scale in a landscape ethnoecological partitioning of a mountainous landscape (Gyimes, Eastern Carpathians, Romania). J. Ethnobiol. Ethnomed..

[34] Babai D., Molnár Z. (2014). Small-scale traditional management of highly species-rich grasslands in the Carpathians. Agric. Ecosyst. Environ..

[35] Biró É., Babai D., Bódis J., Molnár Z. (2014). Lack of knowledge or loss of knowledge? Traditional ecological knowledge of population dynamics of threatened plant species in East-Central Europe. J. Nat. Conserv..

[36] Kis S., Molnár V.A. (2025). Content Analysis of Digital Archives Contributes to the Historical Distribution and Folk Knowledge of the Highly Toxic *Cicuta virosa* L. in Hungary. Plants.

[37] Micu D.M., Dumitrescu A., Cheval S., Birsan M.V. (2016). Climate of the Romanian Carpathians.

[38] Newing N., Eagle C., Puri R., Watson C. (2011). Conducting Research in Conservation: A Social Science Perspective.

[39] R Core Team (2026). R: A Language and Environment for Statistical Computing.

[40] Brooks M.E., Kristensen K., van Benthem K.J., Magnusson A., Berg C.W., Nielsen A., Skaug H.J., Maechler M., Bolker B.M. (2017). glmmTMB balances speed and flexibility among packages for zero-inflated generalized linear mixed modeling. R. J..

[41] McGillycuddy M., Popovic G., Bolker B.M., Warton D.I. (2025). Parsimoniously fitting large multivariate random effects in glmmTMB. J. Stat. Softw..

[42] Hartig F. (2024). DHARMa: Residual Diagnostics for Hierarchical (Multi-Level/Mixed) Regression Models. https://CRAN.R-project.org/package=DHARMa.

[43] Lüdecke D., Ben-Shachar M.S., Patil I., Waggoner P., Makowski D. (2021). performance: An R package for assessment, comparison and testing of statistical models. J. Open Source Softw..

[44] Ben-Shachar M.S., Lüdecke D., Makowski D. (2020). effectsize: Estimation of effect size indices and standardized parameters. J. Open Source Softw..

[45] Ellen R. (1993). The Cultural Relations of Classification: An Analysis of Nualu Animal Categories from Central Seram.

[46] Gómez-Baggethun E., Corbera E., Reyes-García V. (2013). Traditional ecological knowledge and global environmental change: Research findings and policy implications. Ecol. Soc..

[47] Cribb P.J., Kell S.P., Dixon K.W., Barrett R.L., Dixon K.W., Kell S.P., Barrett R.L., Cribb P.J. (2003). Orchid conservation: A global perspective. Orchid Conservation.

[48] Maffi L. (2005). Linguistic, cultural, and biological diversity. Annu. Rev. Anthropol..

[49] Berlin B. (1992). Ethnobiological Classification: Principles of Categorization of Plants and Animals in Traditional Societies.

[50] Atran S. (1993). Cognitive Foundations of Natural History: Towards an Anthropology of Science.

[51] Reyes-García V., Guèze M., Luz A.C., Paneque-Gálvez J., Macía M.J., Orta-Martínez M., Pino J., Rubio-Campillo X. (2013). Evidence of traditional knowledge loss among a contemporary indigenous society. Evol. Hum. Behav..

[52] Pretty J., Adams B., Berkes F., de Athayde S.F., Dudley N., Hunn E., Maffi L., Milton K., Rapport D., Robbins P. (2009). The intersections of biological diversity and cultural diversity: Towards integration. Conserv. Soc..

[53] Csergő A.M., Demeter L., Turkington R. (2013). Declining diversity in abandoned grasslands of the Carpathian Mountains: Do dominant species matter?. PLoS ONE.

[54] Loos J., Gallersdörfer J., Hartel T., Dolek M., Sutcliffe L. (2021). Limited effectiveness of EU policies to conserve an endangered species in high nature value farmland in Romania. Ecol. Soc..

[55] Molnár Z., Babai D. (2021). Inviting ecologists to delve deeper into traditional ecological knowledge. Trends Ecol. Evol..

[56] Molnár Z., Aumeeruddy-Thomas Y., Babai D., Díaz S., Garnett S.T., Hill R., Bates P., Brondízio E.S., Cariño J., Demeter L. (2024). Towards richer knowledge partnerships between ecology and ethnoecology. Trends Ecol. Evol..

[57] International Society of Ethnobiology Code of Ethics (with 2008 Additions). http://ethnobiology.net/code-of-ethics.

[58] General Data Protection Regulation (EU) 2016/679 of the European Parliament and of the Council. https://gdpr-info.eu/.

